# Formulation and Evaluation of Floating Oral *In Situ* Gelling System of Amoxicillin

**DOI:** 10.5402/2011/276250

**Published:** 2011-07-28

**Authors:** Dasharath M. Patel, Divyesh K. Patel, Chhagan N. Patel

**Affiliations:** Department of Pharmaceutics, Shri Sarvajanik Pharmacy College, Near Arvind Baug, Mehsana, Gujarat 384 001, India

## Abstract

*Purpose*. Effective *Helicobacter pylori* eradication requires delivery of the antibiotic locally in the stomach. High dose of amoxicillin (750 to 1000 mg) is difficult to incorporate in floating tablets but can easily be given in liquid dosage form. Keeping the above facts in mind, we made an attempt to develop a new floating *in situ* gelling system of amoxicillin with increased residence time using sodium alginate as gelling polymer to eradicate *H. pylori*. *Methods*. Floating *in situ* gelling formulations were prepared using sodium alginate, calcium chloride, sodium citrate, hydroxypropyl methyl cellulose K100, and sodium bicarbonate. The prepared formulations were evaluated for solution viscosity, floating lag time, total floating time, and *in vitro* drug release. The formulation was optimized using a 3^2^ full factorial design. Dissolution data were fitted to various models to ascertain kinetic of drug release. Regression analysis and analysis of variance were performed for dependent variables. *Results*. All formulations (*F*_1_–*F*_9_) showed floating within 30 s and had total floating time of more than 24 h. All the formulations showed good pourability. It was observed that concentration of sodium alginate and HPMC K100 had significant influence on floating lag time, cumulative percentage drug release in 6 h and 10 h. The batch *F*_8_ was considered optimum since it showed more similarity in drug release (*f*_2_ = 74.38) to the theoretical release profile. *Conclusion*. Floating in situ gelling system of amoxicillin can be formulated using sodium alginate as a gelling polymer to sustain the drug release for 10 to 12 h with zero-order release kinetics.

## 1. Introduction

*Helicobacter pylori *(*H. pylori*) is one of the most common pathogenic bacterial infections. It is associated with the development of serious gastroduodenal disease, including peptic ulcers, gastric lymphoma, and acute chronic gastritis. *H*. *pylori *resides mainly in the gastric mucosa or at the interface between the mucous layer and the epithelial cells of the antral region of the stomach. Antibiotics required for eradication of *H. pylori* are high in dose and in more frequencies [1]. This is because of the low concentration of the antibiotic reaching the bacteria under the mucosa, instability of the drug in the low pH of gastric fluid, and short residence time of the antibiotic in the stomach, leading to incomplete eradication of *H. pylori.*

Amoxicillin is a semisynthetic, orally absorbed, broad-spectrum antibiotic. It is widely used in a standard eradication treatment of gastric *H. pylori *infection combined with a second antibiotic and an acid-suppressing agent [2]. As conventional drug delivery systems do not remain in the stomach for prolonged periods, they are unable to deliver the amoxicillin to the site of infection in effective concentrations. Therefore, it is necessary to design drug delivery systems that not only alleviate the shortcomings of conventional delivery vehicles but also deliver amoxicillin to the infected cell lines. Some researchers had prepared and reported new amoxicillin formulations, such as floating tablets [3, 4], mucoadhesive tablets [5], and mucoadhesive microspheres [6], which were able to reside in stomach for an extended period for more effective *H. pylori *eradication. Amongst the described formulations, the floating tablet is preferred for better and less variable gastric retention, but it has a limitation of incorporation of high dose of the drug. The drug with high dose like amoxicillin can be easily incorporated in liquid *in situ* gelling formulation that upon oral administration can float for a prolonged period of time in the stomach.

Keeping the above facts in mind, we made an attempt to develop a new floating *in situ* gelling system of amoxicillin with increased residence time using sodium alginate as gelling polymer [7] and hydroxypropyl methyl cellulose K100 (HPMC K100) as thickening agent [8] with a potential of *H. Pylori *eradication. The proposed new sodium alginate-based amoxicillin floating *in situ* gelling systems would have the advantage of ease of administration, as being a liquid, and also be more patient compliant.

## 2. Materials and Methods

### 2.1. Materials

Amoxicillin and hydroxypropyl methylcellulose K100 (HPMC K100) were purchased from Yarrow Chem. Products, Mumbai, India. Sodium alginate was obtained from Finar Chemicals Pvt. Ltd, Ahmedabad, India. Sodium bicarbonate was obtained from Shakti Chemicals, Mehsana, India. Calcium chloride and sodium alginate were obtained from Chemdyes Corporation, Ahmedabad, India. All other materials and chemicals used were of either pharmaceutical or analytical grade.

### 2.2. Methods

#### 2.2.1. Preparation of In Situ Gelling Solution

Sodium alginate, at different concentrations (2% w/v, 2.5% w/v, and 3% w/v), was prepared in deionised water containing calcium chloride (0.15% w/v) and sodium citrate (0.5% w/v). HPMC K100 (0.3% w/v, 0.6% w/v, and 0.9% w/v) was added to it. The sodium alginate solution was heated to 50°C with stirring. After cooling below 40°C, 1.5% w/v of calcium carbonate and drug were added and dispersed well with continuous stirring. The resulting sodium alginate *in situ* gel solution containing amoxicillin was finally stored in amber colour narrow mouth bottles until further use.

#### 2.2.2. Measurement of Viscosity of In Situ Gelling Solution

The viscosities of the prepared solutions were determined by brook field viscometer (Brookfield viscometer, model-LVDV-II pro, USA). The samples (100 mL) were sheared at a rate of 100 r/min using suitable spindle at room temperature. Viscosity measurement for each sample was done in triplicate, with each measurement taking approximately 30 s.

#### 2.2.3. In Vitro Gelation Study

Gelation of *in situ* gelling solution was carried out by taking 500 mL of 0.1 N hydrochloric acid (HCl, pH 1.2) in a beaker. Accurately measured 10 mL of solution was added to HCl with mild agitation that avoids breaking of formed gel. Gelling was observed visually by qualitative measurement.

#### 2.2.4. In Vitro Floating Study

Floating study of *in situ* gelling solution was carried out in 500 mL of 0.1 N HCl (pH 1.2) in a beaker. Accurately measured 10 mL of solution was added to HCl with mild agitation. Time required for floating on surface after adding solution (floating lag time) and total floating time were measured [9, 10].

#### 2.2.5. In Vitro Drug Release Study

The *in vitro* release rate of amoxicillin from sustained release *in situ* gel was performed using USP apparatus [11] (model TDT-08T, Electrolab, Mumbai, India) fitted with paddle (50 r/min) at 37 ± 0.5°C using 500 mL of 0.1 N HCl as a dissolution medium. This speed was slow enough to avoid the breaking of gelled formulation and was maintaining the mild agitation conditions believed to exist *in vivo*. At the predetermined time intervals, 10 mL samples were withdrawn, filtered through a 0.45 *μ*m membrane filter, diluted, and assayed at 272 nm using a Shimadzu UV 1800 double-beam spectrophotometer (Shimadzu, Kyoto, Japan). Cumulative percentage drug release (CPR) was calculated using an equation obtained from a calibration curve.

#### 2.2.6. Preliminary Screening

The preliminary screening was performed to optimize amount of calcium chloride and sodium bicarbonate in the formulation. For optimization of amount of calcium chloride, *in situ* gelling systems of amoxicillin were prepared using different concentration of calcium chloride and proportionally sodium citrate as shown in Table 1. Prepared *in situ* gelling systems were tested for gelling strength and CPR at 6 h. For optimization of amount of sodium bicarbonate, *in situ* gelling systems of amoxicillin were prepared using different concentration of sodium bicarbonate as shown in Table 2. Prepared *in situ* gelling systems were tested for floating lag time (FLT) and CPR at 6 h. 

#### 2.2.7. Optimization of Variables Using Full Factorial Design

A 3^2^ randomized full factorial design was used in the present study. In this design, 2 factors were evaluated, each at 3 levels, and experimental trials were performed for all 9 possible combinations [12]. The concentration of sodium alginate (*X*_1_) and concentration of HPMC K100 (*X*_2_) were chosen as independent variables in 3^2^ full factorial design, while FLT (floating lag time), *Q*_1_, *Q*_6_, and *Q*_10_ (% drug release after 1, 6, and 10 hours, resp.) were taken as dependent variables. The formulation layout for the factorial design batches (*F*_1_–*F*_9_) is shown in Table 3.

#### 2.2.8. Kinetic Modeling of Dissolution Data

The dissolution profile of all batches were fitted to various models such as zero order, first order, Higuchi [13], Hixon and Crowell [14], and Korsemeyer et al. [15], to ascertain the kinetic of drug release. The method described by Korsemeyer et al. was used to describe the mechanism of drug release.

#### 2.2.9. Comparison of Release Profiles for Selection of Optimum Batch

The similarity factor (*f*_2_) given by SUPAC guidelines for a modified release dosage form was used as a basis to compare dissolution profiles. The dissolution profiles are considered to be similar when *f*_2_ is between 50 and 100. The dissolution profiles of products were compared using an *f*_2_ which is calculated from the following formula:

(1) $$f_{2}=50\times \log\left\{{\left[1+\left(\frac{1}{n}\right){\overset{n}{\underset{t=1}{\sum }}w_{t}\left(R_{t}-T_{t}\right)}^{2}\right]}^{-0.5}\times 100\right\},$$

where *n* is the dissolution time, and *R*_*t*_ and *T*_*t*_ are the reference (here, this is the theoretical dissolution profile of amoxicillin) and test dissolution value at time *t* [16]. 

#### 2.2.10. Fourier Transform Infrared Spectroscopy

Fourier transform infrared (FTIR) spectra of amoxicillin, a physical mixture of amoxicillin-HPMC K100, and a physical mixture of amoxicillin-sodium alginate were recorded using KBr mixing method on FTIR instrument available at central instrument laboratory of the institute (FTIR-1700, Shimadzu, Kyoto, Japan).

## 3. Results and Discussion

### 3.1. Results of Preliminary Screening

Three batches (*I*-1, *I*-2, and *I*-3) were prepared to optimize amount of calcium chloride and proportionally sodium citrate. From the observation of gelling strength and CPR at 6 h of prepared batches as shown in Table 1, it was concluded that only batch *I*-3 (containing 0.15% w/v of calcium chloride) formed the closed network stiff gel and controlled the release of drug for more than 6 h. 

Three batches (*C*-1, *C*-2, and *C*-3) were prepared to optimize amount of sodium bicarbonate (NaHCO_3_). From the observation of floating lag time and CPR at 6 h of prepared batches as shown in Table 2, it was concluded that batch *C*-2 (containing 1.5% NaHCO_3_) had optimum floating lag time (48 s) and CPR at 6 h (71.57). 

### 3.2. Full Factorial Design

A statistical model incorporating interactive and polynominal terms was used to evaluate the responses

(2) $$Y=b_{0}+b_{1}X_{1}+b_{2}X_{2}+b_{12}X_{1}X_{2}+b_{11}X_{1}X_{1}+b_{22}X_{2}X_{2},$$

where *Y* is the dependent variable, *b*_0_ is the arithmetic mean response of the 9 runs, and *b*_1_ is the estimated coefficient for the factor *X*_*i*_. The main effects (*X*_1_ and *X*_2_) represent the average result of changing 1 factor at a time from its low to high values. The interaction terms (*X*_1_ *X*_2_) show how the response changes when two factors are simultaneously changed. The polynomial terms (*X*_1_^2^ and *X*_2_^2^) are included to investigate nonlinearity. The release profile for 9 batches showed a variation (i.e., initial 1 hr release ranging from 41.90% to 48.39% and drug released after 10 hr ranging from 83.97% to 100.37%). The data indicate that the release profile of the drug is strongly dependent on the selected independent variables. The fitted equations (full and reduced) relating the responses, FLT, *Q*_1_, *Q*_6_, and *Q*_10_, to the transformed factor are shown in the Table 4. The polynomial equations can be used to draw conclusions after considering the magnitude of coefficient and the mathematical sign it carries (i.e., negative or positive). Table 4 shows the results of analysis of variance (ANOVA), which was performed to identify insignificant factors. Data were analyzed using Microsoft Excel.

*R* ^2^ value for FLT, *Q*_1_, *Q*_6_, and *Q*_10_ is 0.87, 0.9956, 0.935, and 0.92, respectively, indicating good correlation between dependent and independent variables. The reduced models were developed for response variables by omitting the insignificant terms with *P* > 0.05. The terms with *P* < 0.05 were considered statistically significant and retained in the reduced model. The coefficients for full and reduced models for response variables are shown in Table 4. 

### 3.3. Full and Reduced Model for FLT

The significance levels of the coefficients *b*_1_, *b*_12_, *b*_11_, and *b*_22_ were found to be *P* = 0.255, 1, 0.478, and 0.603, respectively, so they were omitted from the full model to generate a reduced model. The results of statistical analysis are shown in Table 4. The coefficients *b*_0_ and *b*_2_ were found to be significant at *P* < 0.05; hence, they were retained in the reduced model. The reduced model was tested in proportion to determine whether the coefficients *b*_1_, *b*_12_, *b*_11_, and *b*_22_ contribute significance information to the prediction of FLT [17]. The results of model testing are shown in Table 5. The critical value of *F* for *α* = 0.05 is equal to 9.12 (df = 4,3). Since the calculated value (*F* = 0.73) is less than critical value (*F* = 9.12), it may be concluded that the interaction terms *b*_1_, *b*_12_, *b*_11_, and *b*_22_ do not contribute significantly to the prediction of FLT and can be omitted from the full model to generate the reduced model.

### 3.4. Full and Reduced Model for *Q*_6_

The significance levels of the coefficients *b*_2_, *b*_12_, *b*_11_, and *b*_22_ were found to be *P* = 0.058, 0.236, 0.291, and 0.443, respectively, so they were omitted from the full model to generate a reduced model. The results of statistical analysis are shown in Table 4. The coefficients *b*_0_ and *b*_1_ were found to be significant at *P* < 0.05; hence, they were retained in the reduced model. The reduced model was tested in proportion to determine whether the coefficients *b*_2_, *b*_12_, *b*_11_, and *b*_22_ contribute significance information to the prediction of *Q*_6_. The results of model testing are shown in Table 5. The critical value of *F* for *α* = 0.05 is equal to 9.12 (df = 4,3). Since the calculated value (*F* = 3.36) is less than critical value (*F* = 9.12), it may be concluded that the interaction terms *b*_2_, *b*_12_, *b*_11_, and *b*_22_ do not contribute significantly to the prediction of *Q*_6_ and can be omitted from the full model to generate the reduced model.

### 3.5. Full and Reduced Model for *Q*_10_

The significance levels of the coefficients *b*_2_, *b*_12_, *b*_11_, and *b*_22_ were found to be *P* = 0.120, 0.104, 0.109, and 0.403, respectively, so they were omitted from the full model to generate a reduced model. The results of statistical analysis are shown in Table 4. The coefficients *b*_0_ and *b*_1_ were found to be significant at *P* < 0.05; hence, they were retained in the reduced model. The reduced model was tested in proportion to determine whether the coefficients *b*_2_, *b*_12_, *b*_11_, and *b*_22_ contribute significance information to the prediction of *Q*_10_. The results of model testing are shown in Table 5. The critical value of *F* for *α* = 0.05 is equal to 9.12 (df = 4,3). Since the calculated value (*F* = 3.99) is less than critical value (*F* = 9.12), it may be concluded that the interaction terms *b*_2_, *b*_12_, *b*_11_, and *b*_22_ do not contribute significantly to the prediction of *Q*_10_ and can be omitted from the full model to generate the reduced model.

### 3.6. Kinetic Modeling of Dissolution Data

The kinetics of the dissolution data were well fitted to zero order, Higuchi model, and Korsemeyer-Peppas model as evident from regression coefficients (Table 6). In case of the controlled or sustained release formulations, diffusion, swelling, and erosion are the three most important rate controlling mechanisms. Formulations containing swelling polymers show swelling as well as diffusion mechanism because the kinetic of swelling includes relaxation of polymer chains and imbibitions of water, causing the polymer to swell and changing it from a glassy to rubbery state. The diffusion exponent *n* is the indicative of the mechanism of drug release from the formulation. For a swellable cylindrical drug delivery system, the *n* value of less than 0.45 is indicative of Fickian diffusion-ontrolled drug release, *n* value between 0.45 and 0.89 signifies anomalous (non-Fickian) transport, *n* value of 0.89 indicates case II transport, and *n* value greater than 0.85 indicates super case II transport [18, 19]. The value of diffusion exponent *n* for all factorial formulations is less than 0.45 (Table 6) indicating Fickian drug release from the formulations. 

### 3.7. Comparison of Release Profiles for Selection of Optimum Batch

**Figure 1:**
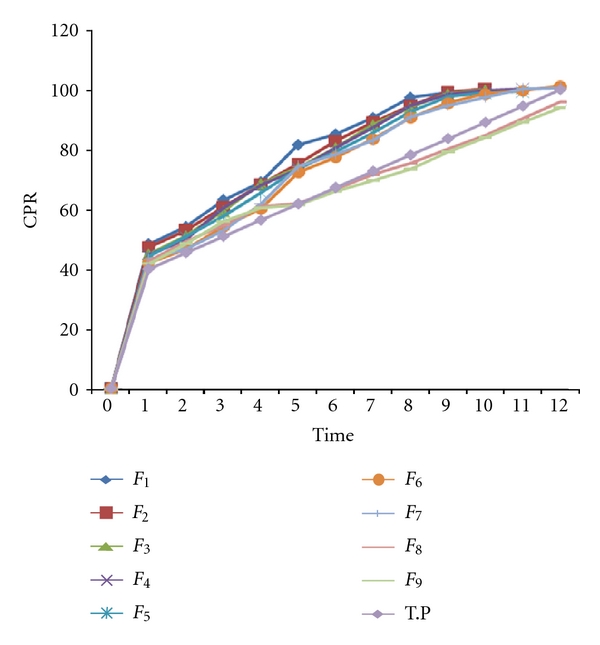
Comparison of dissolution profile.

The release profiles of the developed formulations are shown in Figure 1. The values of similarity factor (*f*_2_) for batches *F*_5_ to *F*_9_ were greater than 50 compared with theoretical release profile (Table 3) indicating good similarity in dissolution. The batch *F*_8_ showed maximum value of *f*_2_ (74.38) and hence was selected as optimum batch. 

### 3.8. Fourier Transform Infrared Spectroscopy

**Figure 2:**
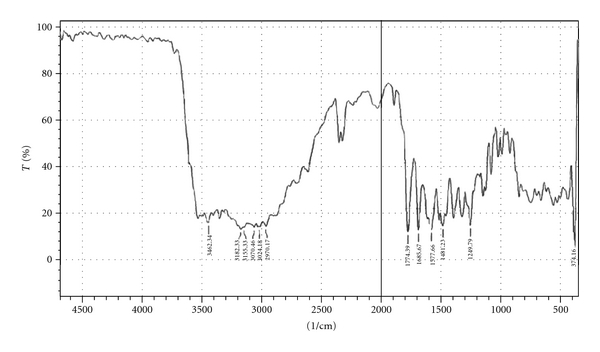
FTIR spectrum of amoxicillin.

**Figure 3:**
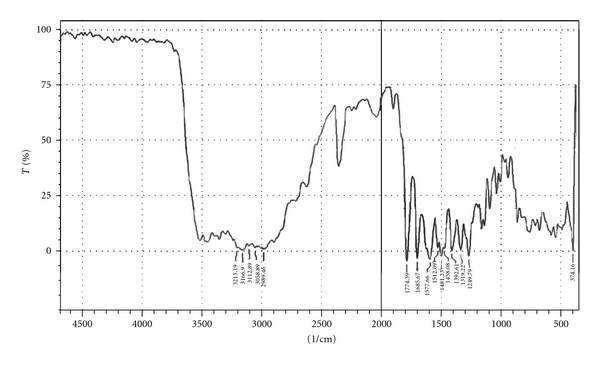
FTIR spectrum of amoxicillin and HPMC K100.

**Figure 4:**
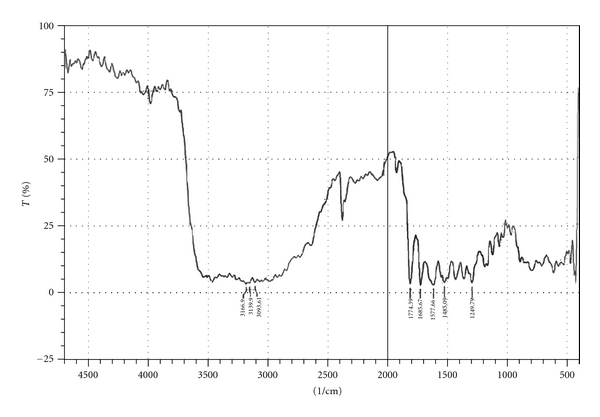
FTIR spectrum of amoxicillin and HPMC K100.

Drug-excipients interactions play a vital role in the release of drug from formulation. Fourier transform infrared spectroscopy has been used to study the physical and chemical interactions between drug and the excipients used. The pure amoxicillin and its mixture with sodium alginate and HPMC K100 were mixed separately with IR grade KBr and were scanned over a range of 400–4500 cm^−1^ using FTIR instrument (FTIR-1700, Shimadzu, Kyoto, Japan). The drug exhibits peak due to ketonic, primary amine, secondary amine, and hydroxyl (broad) group. It was observed that there were no changes in these main peaks in the IR spectra of a mixture of drug and polymers (Figures 2–4). The FTIR study revealed no physical or chemical interactions of amoxicillin with sodium alginate and HPMC K100 as evident from Figures 3 and 4.

## 4. Conclusion

Floating *in situ* gelling system of amoxicillin with increased gastric residence time can be formulated using sodium alginate as a gelling polymer and HPMC K100 as a thickening agent. The prepared formulation can provide a site-specific delivery of amoxicillin for 10 to 12 h with zero-order release kinetics. 

## Figures and Tables

**Table 1:** Formulation of amoxicillin *in situ* gel using different concentrations of calcium chloride and sodium citrate.

Name of ingredient	Quantity in 100 mL (% w/v)		
	*I*-1	*I*-2	**I**-3
Amoxicillin	7.5	7.5	7.5
Calcium chloride	0.075	0.10	0.15
Sodium citrate	0.25	0.33	0.5
Sodium alginate	2.5	2.5	2.5
HPMC K100	0.8	0.8	0.8
Sodium bicarbonate	1.5	1.5	1.5
Deionised water	Q.S	Q.S	Q.S
Gelling strength	Open network gel	Open network gel	Close network Stiff gel
CPR	100 (in 3 h)	100 (in 5 h)	70.14 (in 6 h)

CPR indicates cumulative percentage release; HPMC K100 indicates hydroxypropyl methyl cellulose K100; Q.S indicates quantity sufficient.

**Table 2:** Formulation of amoxicillin *in situ* gel using different concentrations of sodium bicarbonate.

Name of ingredient	Quantity in 100 mL (% w/v)		
	*C*-1	*C*-2	*C*-3
Amoxicillin	7.5	7.5	7.5
Calcium chloride	0.15	0.15	0.15
Sodium citrate	0.5	0.5	0.5
Sodium alginate	2.5	2.5	2.5
HPMC K100	0.8	0.8	0.8
Sodium bicarbonate	1	1.5	2
Deionised water	Q.S	Q.S	Q.S
Floating lag time (s)	55	48	30
CPR at 6 h (%)	67.50	71.57	82.84

CPR indicates cumulative percentage release; HPMC K100 indicates hydroxypropyl methyl cellulose K100; Q.S indicates quantity sufficient.

**Table 3:** Formulation and evaluation of batches in 3^2^ full factorial design.

Batch code	Variable levels in coded form		FLT (s)	*Q*_1_ (%)	*Q*_6_ (%)	*Q*_10_ (%)	*f*_2_	Viscosity (cps)
	*X*_1_	*X*_2_					
*F*_1_	−1	−1	18	48.39	85.04	100.24	42.78	171.1
*F*_2_	−1	0	15	47.41	82.82	100.37	45.51	186.3
*F*_3_	−1	1	13	44.92	80.57	99.93	47.86	193.7
*F*_4_	0	−1	21	44.92	80.57	99.93	48.01	211.3
*F*_5_	0	0	15	44.21	79.16	98.80	51.11	230.4
*F*_6_	0	1	10	41.99	77.55	98.61	54.48	356.7
*F*_7_	1	−1	16	43.23	78.55	97.55	52.88	431.9
*F*_8_	1	0	12	42.88	67.03	85.02	74.38	542.1
*F*_9_	1	1	11	41.90	65.99	83.97	70.18	634.3

Coded values	Actual values							
	*X*_1_	*X*_2_						

−1	2%	0.3%						
0	2.5%	0.6%						
1	3%	0.9%						

All batches contained 7.5% of amoxicillin, 1.5% of sodium bicarbonate, 0.15% of calcium chloride, and 0.5% of sodium citrate. *X*_1_ indicates concentration of sodium alginate, *X*_2_ concentration of HPMC K100. *Q*_1_, *Q*_6_, and *Q*_10_ indicate percentage drug released after 1, 6, and 10 h, respectively. FLT indicates floating lag time; *f*_2_ indicates similarity factor.

**Table 4:** Summary of results of regression analysis.

	*b*_0_	*b*_1_	*b*_2_	*b*_12_	*b*_11_	*b*_22_
For FLT						

Response (FLT)						
FM	14.77	−1.16	−3.5	−3.4∗10^−16^	−1.16	0.83
RM	14.56	—	−3.5	—	—	—

For *Q*_1_						

Response (*Q*_1_)						
FM	44.11	−2.12	−1.29	0.53	1.09	−0.69
RM	—	—	—	—	—	—

For *Q*_6_						

Response (*Q*_6_)						
FM	77.95	−6.14	−3.34	−2.02	−2.42	1.70
RM	77.47	−6.14	—	—	—	—

For *Q*_10_						

Response (*Q*_10_)						
FM	97.79	−5.66	−2.53	−3.31	−4.6	1.97
RM	96.04	−5.66	—	—	—	—

FM= Full model, RM= Reduced model; *Q*_1_, *Q*_6_, and *Q*_10_ indicate percentage of drug released after 1, 6, and 10 h, respectively; FLT indicates floating lag time.

**Table 5:** Calculations for testing the model in portions.

	DF	SS	MS	*F*	*R* ^2^	
For floating lag time						

Regression						
FM	5	85.77	17.15	4.13	0.87	*F* calc. = 0.73 *F* table = 9.12 DF = (4,3)
RM	1	73.5	73.5	20.81	0.002	
Error						
FM	3	12.44	4.15			
RM	7	24.72	3.53			

For *Q*_6_						

Regression						
FM	5	327.42	65.48	8.71	0.935	*F* calc. = 3.36 *F* table = 9.12 DF = (4,3)
RM	1	226.44	226.44	12.83	0.64	
Error						
FM	3	22.54	7.51			
RM	7	123.51	17.64			

For *Q*_10_						

Regression						
FM	5	325.37	65.07	7.83	0.92	*F* calc. = 3.99 *F* table = 9.12 DF = (4,3)
RM	1	192.66	192.66	—	0.55	
Error						
FM	3	12.44	4.15			
RM	7	24.72	3.53			

DF indicates the degree of freedom; SS: Sum of squares; MS: Mean of squares; *R* ^2^: Regression coefficient; FM: Full model; RM, Reduced model. *Q*_6_ and *Q*_10_ indicate percentage of drug released after 6 and 10 h, respectively.

**Table 6:** Kinetic treatment of dissolution data.

	*F*_1_	*F*_2_	*F*_3_	*F*_4_	*F*_5_	*F*_6_	*F*_7_	*F*_8_	*F*_9_
Zero order									
*b*	5.17	5.27	5.42	5.45	5.44	5.89	5.78	4.59	4.42
*a*	47.48	45.72	43.66	43.52	42.24	37.46	38.33	40.13	40.10
*R* ^2^	0.9619	0.9713	0.9716	0.9692	0.9771	0.9844	0.9811	0.9969	0.9941
First order									
*b*	0.030	0.031	0.033	0.033	0.033	0.036	0.035	0.029	0.029
*a*	1.699	1.686	1.668	1.667	1.657	1.619	1.626	1.637	1.634
*R* ^2^	0.9465	0.9554	0.9527	0.9496	0.9604	0.9702	0.9666	0.9874	0.9812
Higuchi									
*b*	24.07	24.42	25.13	25.31	25.14	27.02	26.50	20.73	20.04
*a*	22.88	20.91	18.09	17.74	16.81	10.41	11.76	19.70	20.54
*R* ^2^	0.9832	0.9880	0.9892	0.9877	0.9905	0.9893	0.9873	0.9897	0.9901
Hixon Crowell									
*b*	−0.09	−1.75	−1.80	−1.81	−1.83	−1.96	−1.92	−1.52	−1.47
*a*	0.973	18.09	18.77	18.82	19.25	20.84	20.55	19.95	19.97
*R* ^2^	−0.9525	−0.9713	−0.9715	−0.9691	−0.9775	−0.9843	−0.9810	−0.9969	−0.9940
Korsemeyer and Peppas									
*n*	0.333	0.341	0.361	0.364	0.363	0.396	0.387	0.316	0.313
*a*	−0.34	−0.355	−0.376	−0.378	−0.385	−0.423	−0.416	−0.395	−0.399
*R* ^2^	0.9845	0.9857	0.9885	0.9868	0.9877	0.9802	0.9756	0.9832	0.9859

*b* = slope, *a* = intercept, *R* ^2^ = Square of correlation coefficient, and *n* = diffusion exponent.
